# Supplementary material for: A TLR4 Agonist Induces Osteosarcoma Regression by Inducing an Antitumor Immune Response and Reprogramming M2 Macrophages to M1 Macrophages
Source: Cancers (Basel). 2023 Sep 19;15(18):4635. doi: 10.3390/cancers15184635 (PMC10526955; doi:10.3390/cancers15184635)
Supplement: Supplementary file 1 [file cancers-15-04635-s001.zip › cancers-2544780-supplementary.pdf]

A

B

C

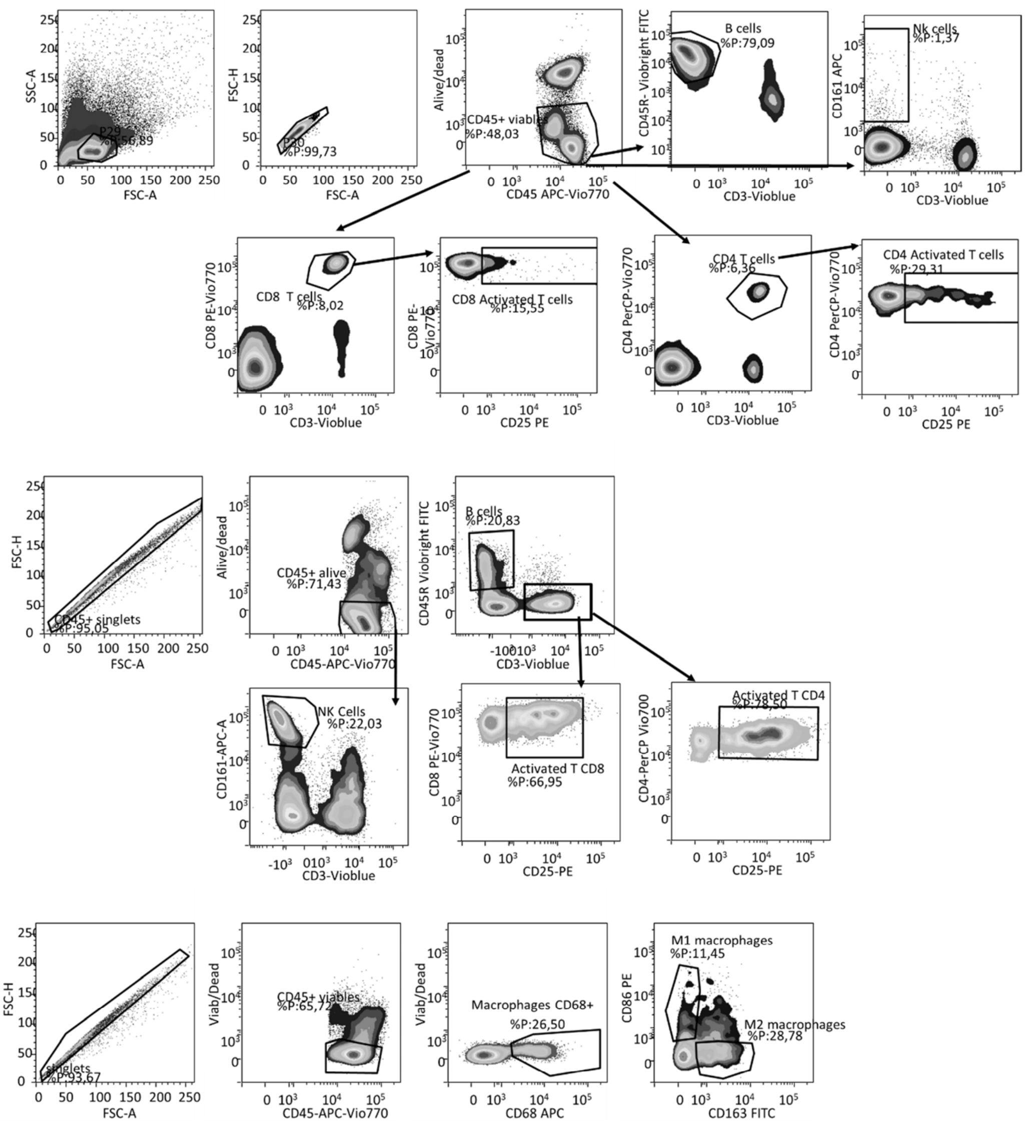


**Figure S1: Gating strategy used to phenotype the different immune populations by flow cytometry**. (A) Gating strategy used to identify, B cells, NK cells, CD4 Activated T cells, CD8 Activated T cells from the spleen. (B) Gating strategy used to identify, B cells, NK cells, CD4 Activated T cells, CD8 Activated T cells from tumors infiltrating immune cells. (C) Gating strategy used to identify total macrophages, M1 macrophages and M2 macrophages.

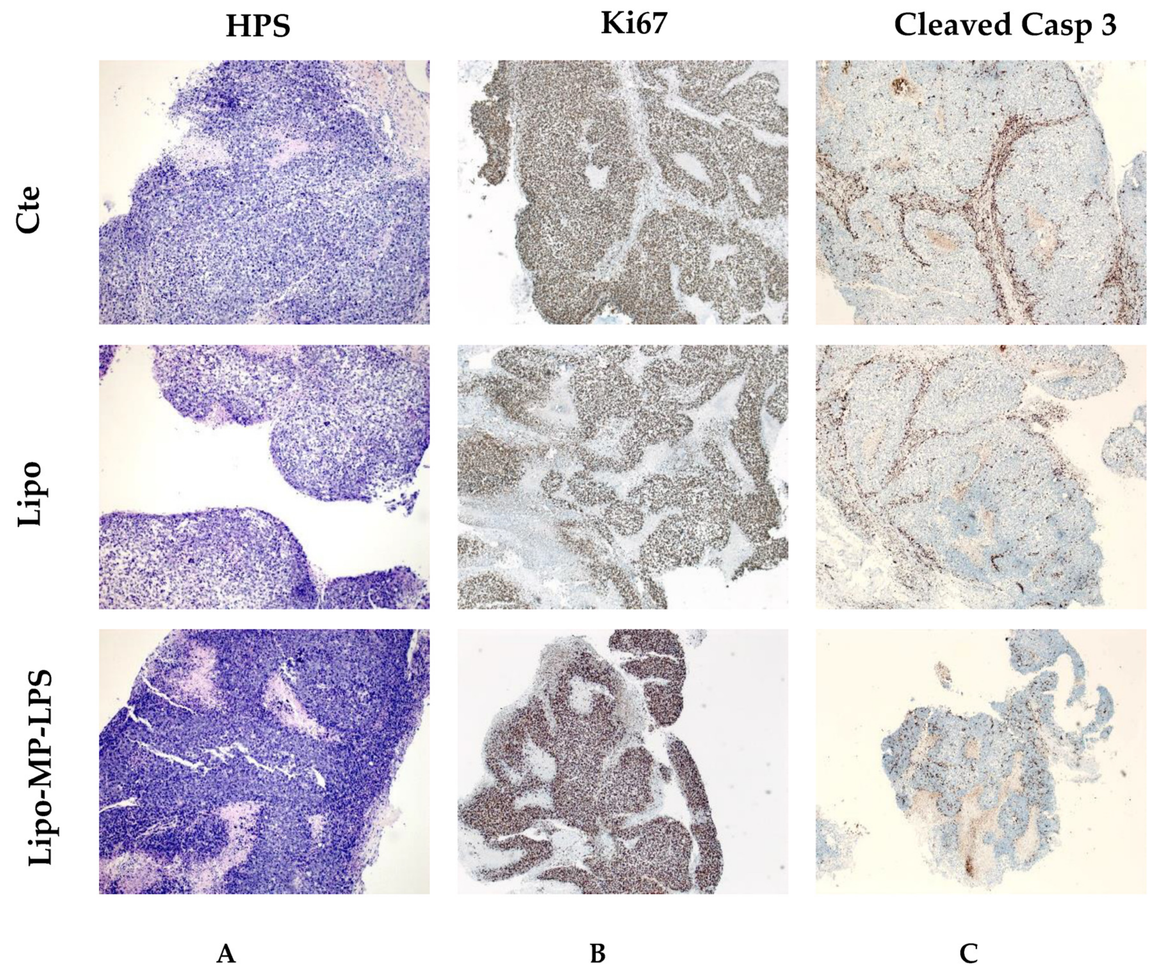


**Figure S2: Lipo-MP-LPS effect on organotypic model of OsA**. (A) HPS Staining of tumors. LipoMP-LPS did not modify the histological composition of tumors. (B) Immunostaining of Ki67. LipoMP-LPS treatment did not impact on the rate of proliferative Ki67+ tumor cells. (C) Immunostaining of cleaved caspace 3. LipoMP-LPS treatment did not increase the rate of cleaved caspase 3 cells indicating the absence of direct effect of LipoMP-LPS on OsA cells (Cte:273/FOV, Lipo:205/FOV, LipoMP-LPS:189/FOV).

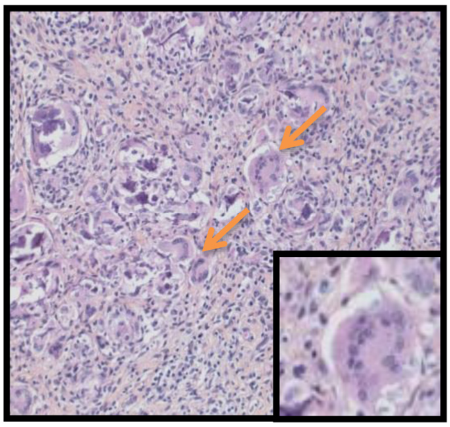

**Figure S3: Detection of multinucleated giant cells (MGC) in LipoMP-LPS treated tumors.** Multinucleated giant cells identified in HPS-stained slides (orange arrow). Location and morphology of these cells is concordant with osteoclasts. Magnification X20 and X40 (right bottom square).

**Table S1. Markers used for flow cytometry analyses**.

| | Fluorochrome | Dilution | Supplier |
|---|---|---|---|
| CD3 | Vioblue | 1/10 | Miltenyi |
| Viability | Viogreen | 1/100 | Miltenyi |
| CD45R | Viobright FITC | 1/10 | Miltenyi |
| CD25 | PE | 1/50 | Biolegend |
| CD4 | PerCP-Vio700 | 1/10 | Miltenyi |
| CD8 | PE-Vio770 | 1/10 | Miltenyi |
| CD161 | APC | 1/10 | Miltenyi |
| CD45 | APC-Vio770 | 1/25 | Miltenyi |
| CD163 | FITC | 1/10 | Biorad |
| CD86 | PE | 1/10 | Miltenyi |
| CD68 | APC | 1/10 | Miltenyi |
